# The association of plasma cystatin C proteoforms with diabetic chronic kidney disease

**DOI:** 10.1186/s12953-016-0096-7

**Published:** 2016-03-25

**Authors:** Hussein N. Yassine, Olgica Trenchevska, Zhiwei Dong, Yara Bashawri, Juraj Koska, Peter D. Reaven, Randall W. Nelson, Dobrin Nedelkov

**Affiliations:** University of Southern California, Los Angeles, CA USA; Molecular Biomarkers Laboratory, Biodesign Institute, Arizona State University, P.O. Box 876601, Tempe, AZ 85287-6601 USA; Phoenix VA Health Care System, Phoenix, AZ USA

**Keywords:** Cystatin C, Truncations, Mass spectrometry, Chronic kidney disease

## Abstract

**Background:**

Cystatin C (CysC) is an endogenous cysteine protease inhibitor that can be used to assess the progression of kidney function. Recent studies demonstrate that CysC is a more specific indicator of glomerular filtration rate (GFR) than creatinine. CysC in plasma exists in multiple proteoforms. The goal of this study was to clarify the association of native CysC, CysC missing N-terminal Serine (CysC des-S), and CysC without three N-terminal residues (CysC des-SSP) with diabetic chronic kidney disease (CKD).

**Results:**

Using mass spectrometric immunoassay, the plasma concentrations of native CysC and the two CysC truncation proteoforms were examined in 111 individuals from three groups: 33 non-diabetic controls, 34 participants with type 2 diabetes (DM) and without CKD and 44 participants with diabetic CKD. Native CysC concentrations were 1.4 fold greater in CKD compared to DM group (*p* = 0.02) and 1.5 fold greater in CKD compared to the control group (*p* = 0.001). CysC des-S concentrations were 1.55 fold greater in CKD compared to the DM group (*p* = 0.002) and 1.9 fold greater in CKD compared to the control group (*p* = 0.0002). CysC des-SSP concentrations were 1.8 fold greater in CKD compared to the DM group (*p* = 0.008) and 1.52 fold greater in CKD compared to the control group (*p* = 0.002). In addition, the concentrations of CysC proteoforms were greater in the setting of albuminuria. The truncated CysC proteoform concentrations were associated with estimated GFR independent of native CysC concentrations.

**Conclusion:**

Our findings demonstrate a greater amount of CysC proteoforms in diabetic CKD. We therefore suggest assessing the role of cystatin C proteoforms in the progression of CKD.

**Electronic supplementary material:**

The online version of this article (doi:10.1186/s12953-016-0096-7) contains supplementary material, which is available to authorized users.

## Background

Cystatin C (CysC) is a cysteine proteinase inhibitor belonging to the type 2 cystatin gene family [1–3]. It is a non-glycosylated single chain protein with a molecular weight of 13,343 Da. CysC is produced at a constant rate by nucleated cells, and is freely filtered by the renal glomerulus, therefore, has been used to monitor the progression of chronic kidney disease (CKD) [4].

CKD is defined by a decline of glomerular filtration rate (GFR) to <90 mL/min/1.73 m^2^ and by the presence of kidney damage for at least 3 months [5, 6]. CKD affects 26 million American adults, while millions of others are at increased risk. In clinical practice, estimated GFR (eGFR) is used to evaluate CKD and categorize the disease into different stages (1–5) based on severity. GFR is defined as the volume of plasma that can be completely cleared of a particular substance by the kidneys in a unit of time [7]. Accurate diagnosis and staging of CKD is critical for therapy and clinical implications.

Currently, serum creatinine is used to calculate eGFR. However, eGFR determined by creatinine-based equations can be biased by several factors that are not related to kidney function including age, race, sex, muscle mass, dietary intake and other factors affecting plasma creatinine levels. In a recent study by Stevens et al., creatinine-based estimates of GFR could not accurately detect acute changes in kidney function [8]. In addition, 5 – 10 % of excreted creatinine originates from distal tubule secretion. This excretion increases in response to decreased GFR, making it difficult to accurately detect small to moderate changes in GFR [2]. Therapies that block distal tubule secretion of creatinine (e.g., trimethoprim, cimetidine, cefoxitin) can also increase serum creatinine levels, and alter the GFR estimation [9].

In a meta-analysis by Shlipak, et al. [10], reclassification of kidney function with CysC improved the prediction of cardiovascular and renal morbidity and mortality. Additionally, in experimental mice models of acute renal failure, CysC was a more sensitive measure of renal insufficiency after bilateral nephrectomy compared to serum creatinine [11]. In a study by Coll et al. [12], serum creatinine levels increased in 92.15 % of patients with impaired renal function compared to serum CysC levels which was increased in 100 % of patients. CysC started increasing at eGFR value of 88 mL/min/1.73 m^2^, while creatinine levels increased at eGFR of 75 mL/min/1.73 m^2^. Furthermore, the study showed that only CysC levels were significantly elevated in hypertensive patients with no evidence of renal failure and minimal proteinuria [12].

CysC undergoes posttranslational modifications in the plasma to form multiple proteoforms [13]. Protein modifications play important roles in biological processes, and can serve as diagnostic indicators of pathological events. Mass spectrometric immunoassay (MSIA) is a high throughput assay well suited to identify and quantify molecular variants and posttranslational modifications of plasma proteins [14, 15]. MSIA involves the isolation of protein moieties from a biological milieu by immobilized antibodies, followed by mass spectrometric detection. We recently reported in a population of 500 healthy adults 3 posttranslationally modified CysC proteoforms: one containing hydroxyproline, and 2 truncated proteoforms; one missing N-terminal serine, and one lacking three N-terminal residues [13]. The distribution of CysC proteoforms in CKD is not known. In the present study, we investigate the CysC truncations in non-diabetic controls and patients with DM and CKD using MSIA.

## Methods

### Clinical samples

The study was approved by the University of Arizona Institutional Review Board, and written informed consent was obtained from all patients. Three groups of adult participants were recruited: 33 participants without CKD and without DM (control), 34 participants without CKD and with DM (DM group), and 44 participants with both CKD and DM (CKD group). Participants reported to the Center for Clinical and Translational Sciences after an overnight fast. Blood was collected for clinical laboratory measurements (lipid profile, Hemoglobin A1C (HbA1C), C-reactive protein (CRP), and fasting insulin). Additional samples were collected in EDTA tubes, and plasma from these samples was separated and immediately frozen at −80 °C for all other assays. Demographic information (age, sex, ethnicity), physical exam measurements (blood pressure, waist circumference, weight, height, body mass index (BMI)), medication use, and medical history (hypertension, hyperlipidemia, smoking, type and duration of diabetes) were also recorded. GFR was estimated using the Modification of Diet in Renal Disease Study (MDRD) equation. Assignment of CKD (stages 1–5) was based on eGFR levels as described [16]. Exclusion criteria included the following: type 1 diabetes, participation in an active weight loss program, pregnancy, dysuria, thyroid dysfunction, history of cancer, HIV, active infection, other ongoing serious illness or current steroid use. Diabetes classification was based on clinical and medication history, or glycated hemoglobin greater than 6 %.

### Reagents

Polyclonal goat anti-human CysC antibody (Cat. No. GCYS-80A) was obtained from ICLLab (Portland, OR). Affinity purified rabbit anti-bovine beta lactoglobulin antibody (Cat. No. GTX77272) was purchased from GeneTex (Irvine, CA). Native human CysC (Cat. No CRC173B) was obtained from Cell Sciences (Canton, MA). Protein calibration standard I (Cat. No. 206355) was purchased from Bruker (Billerica, MA). Phosphate buffered saline (PBS) buffer (Cat. No. 28372), MES buffered saline (28390), 1, 1’carbonyldiimidazole (97 %) (CDI, 530-62-1), acetonitrile (ACN, A955-4), hydrochloric acid (HCl, A144-212), *N*-Methylpyrrolidinone (NMP, BP1172-4) and affinity pipettes fitted with porous microcolumns (991CUS01) were obtained from Thermo Scientific (Waltham, MA, USA). Beta lactoglobulin (BL) from bovine milk (Cat. No. L8005), Tween 20 (Cat. No. P7949), trifluoroacetic acid (TFA, 299537-100G), sinapic acid (85429-5G), sodium chloride (S7653), HEPES (H3375), ethanolamine (ETA, 398136) and albumin from bovine serum (BSA, A7906), were obtained from Sigma Aldrich (St. Louis, MO, USA). Acetone (Cat. No. 0000017150) was obtained from Avantor Performance Materials (Center Valley, PA, USA).

### Analytical samples preparation

Frozen plasma samples were briefly thawed on ice, centrifuged at 3000 rpm for 5 min, and 50 μL aliquots were placed into low profile 96-well trays. To generate the standard curve, CysC standard was serially diluted in PBS buffer containing 3 g/L BSA, to final concentrations of 1.25 mg/L (standard 1), 0.625 mg/L (standard 2), 0.313 mg/L (standard 3), 0.156 mg/L (standard 4), 0.078 mg/L (standard 5) and 0.039 mg/L (standard 6). Beta lactoglobulin (BL) was used as an internal reference standard (IRS) for quantification, and was prepared in water, to a final concentration of 1 mg/mL. Human plasma samples were diluted 5-times in PBS, 0.1 % Tween prior to analysis. The analytical samples were prepared by combining 20 μL of the CysC standard solution or the diluted plasma with 40 μL of a 1 mg/mL solution of BL, and 100 μL PBS 0.1 % Tween buffer.

### Mass spectrometric immunoassay

Affinity pipettes derivatization and assay execution was done on a Multimek 96-channel pipettor (Beckman Coulter, Brea, CA) as previously described [17] . Initially, affinity pipettes were derivatized with antibodies towards CysC (the targeted protein) and BL (the internal reference standard) in a ratio of 4.5:1 (w/w; CysC:BL, 3.6 μg anti-CysC, 50 μL/well), as described previously in the protocol [13, 17]. Antibody-derivatized pipettes were stored at +4 °C until used. Prior to sample protein extraction, the derivatized affinity pipettes were pre-rinsed with assay buffer (PBS, 0.1 % Tween, 10 aspiration/dispense cycles, 100 μL each). Pipettes were then immersed into a microplate containing analytical samples, and 250 aspirations/dispense cycles were performed (100 μL each) allowing for optimal affinity capture of both CysC and BL. The pipettes were then rinsed with PBS 0.1 % Tween (100 cycles, 100 μL aspiration/dispense volumes), and twice with water (10 cycles and 20 cycles respectively, 100 μL aspiration/dispense each). Protein-loaded tips were then exposed to six-microliter aliquots of MALDI matrix solution (25 g/L sinapic acid in aqueous solution containing 33 % (v/v) acetonitrile, and 0.4 % (v/v) trifluoroacetic acid). After a 10 s delay (to allow for the dissociation of the protein from the capturing antibody), the eluates were dispensed directly onto a 96-well formatted MALDI target. After samples were dried, linear-mode mass spectra was acquired from each sample spot, each consisting of ten thousand laser shots using an *Autoflex* III MALDI-TOF mass spectrometer (Bruker, Billerica, MA). For accurate mass assignment, the mass spectra were externally calibrated with protein standard I (Cat. No. 8206355, Bruker, Billerica, MA) and also with the internal reference standard (mass accuracy up to 0.001 Da). In addition, the mass spectra were baseline subtracted (Tophat algorithm) and smoothed (SavitzkyGolay algorithm; width = 0.2 m/z; cycles = 1), before peak integration with Flex Analysis 3.0 software (Bruker Daltonics). Peak areas for all CysC signals and BL were measured in Zebra software (Beavis Informatics, Ltd.). The hydroxylated proteoforms were not baseline-resolved from their corresponding proteoforms and were therefore co-integrated with their originating proteoforms.

### Quantitative MSIA analysis of CysC proteoforms

**Fig. 1:**
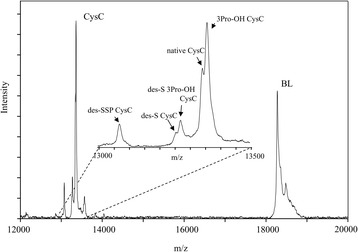
MALDI-MSIA-mass spectra showing all CysC proteoforms. Signals for native CysC along with several cystatin C proteoforms: CysC containing hydroxyproline at position 3 (CysC 3Pro-OH), CysC missing its N-terminal Serine (CysC des-S), and CysC missing its three N-terminal residues (CysC des-SSP) are detected. BL is beta lactoglobulin used as an internal reference standard for quantification

Quantification of CysC was done as previously described [17] . In short, standard curve was generated, utilizing the corresponding protein standard (CysC) and the internal reference standard (BL). Separate standard curves were created with each run, by plotting the ratio of the peak areas of the CysC standard signal and the BL signal (CysC/BL) against CysC standard concentration (c(CysC)). The linear equations obtained were used to calculate the concentrations of native CysC and CysC proteoforms in the analyzed samples, using the ratio of the peak areas of each proteoform to the IRS. Peak area ratios for each CysC proteoforms against BL were summed up, and, total CysC concentration was determined using the standard curve equation. Individual CysC proteoform concentrations were calculated using the percent abundance in correspondence to total CysC. The reproducibility of the assay was tested by analyzing a control sample with known CysC concentration in triplicates with each run. This control sample was used to assess the within and between run variability. MSIA can identify total of 5 CysC proteoforms (Fig. 1). However, due to the inability to be resolved at a baseline level, CysC hydroxylated proteoforms were integrated with their originating proteoforms. An example of a standard curve, together with the corresponding mass spectra is presented in Additional file 1: Figure S1. The control sample run in triplicates showed within-run variability CV of 3.43, and between-run CV of 10.4. The concentrations of the three major CysC proteoforms were analyzed in plasma of all participants using MSIA.

### Statistical analysis

Mean (SD) and median (25th and 75^th^ percentiles) were used to describe normally and non-normally distributed continuous variables, respectively. The control, DM and CKD groups were compared using one-way ANOVA (normally distributed variables) or Kruskal-Wallis test (non-normally distributed variables). Categorical variables were compared using chi-square test. Linear regression was used to analyze the association of GFR with CysC proteoforms. All regression models were adjusted for age, sex, and BMI as covariates. Partial correlation was used to test independent correlation of native CysC, CysC des-SSP and CysC des-S with eGFR. All statistical analyses were performed using SAS version 9.4 software package. For Cystatin C proteoforms, the alpha level was set at 0.02 adjusting for multiple comparisons. For the other variables, the alpha level was set at 0.05.

## Results

The demographic and clinical characteristics of individuals in the control, DM and CKD groups are summarized in Table 1. Participants in the CKD group were significantly older than both the control and DM groups (*p* < 0.001). The DM and CKD groups had elevated BMI (*p* < 0.001), HbA1C (*p* < 0.001) and CRP (*p* < 0.001), as well as decreased levels of HDL cholesterol (*p* < 0.01) compared to controls. As expected, urine microalbumin and serum creatinine were significantly greater, and eGFR was lower in the CKD group compared to the two other groups (*p* < 0.001).

**Table 1:** Demographic, clinical and biochemical characteristics of study participants

Characteristic	N_1_/N_2_/N_3_	Control	DM	CKD	*p*-value
Age, years	33/34/44	43.82 (14.93)	51.97 (11.77)	61.09 (11.79)	<0.001^a,b^
Sex	33/34/44		0.64		
Male		15 (45.45 %)	17 (50 %)	25 (56.82)	
Female		12 (70.59)	17 (50 %)	19 (43.18)	
Race	33/34/44				0.25
Caucasians		24 (72.73 %)	19 (55.88 %)	25 (56.82 %)	
Hispanics		8 (24.24 %)	15 (44.12 %)	16 (36.36 %)	
Others		1 (3.03 %)	0 (0 %)	3 (6.82 %)	
Body mass index, kg/m^2^	33/34/40	24.37 (3.25)	36.33 (10.26)	34.08 (8.28)	<0.001^c^
Waist circumference, cm	32/33/35	86.94 (12.07)	116.52 (20.82)	115.57 (18.95)	<0.001^c^
Glucose, mg/dL	33/34/37	98 (88, 107)	144 (123, 182)	142 (114, 202)	<0.001^c^
Hemoglobin A1C, %	33/31/40	5.2 (4.9, 5.4)	7.5 (6.7, 9.7)	7.6 (6.4, 9.3)	<0.001^c^
LDL cholesterol, mg/dL	33/34/41	118.09 (31.93)	111.09 (39.15)	101.95 (36.98)	0.16
HDL cholesterol, mg/dL	33/34/41	54.97 (16.41)	46.41 (11.60)	43.95 (13.29)	<0.01^c^
Total cholesterol, mg/dL	33/34/41	198.12 (41.58)	191.15 (55.56)	182.15 (43.62)	0.35
Triglycerides, mg/dL	33/34/41	128 (96, 182)	162 (124, 258)	166 (135,268)	0.02^d^
Fasting Insulin, IU	32/23/25	9.53 (7.68)	17.74 (11.32)	16.76 (13.02)	0.01^c^
Urine Microalbumin, mg/mg creatinine	33/33/38	5 (5, 9)	11 (5, 27)	25 (9, 174)	<0.001^c^
CRP, mg/dL	16/24/33	1.24 (0.35, 3.77)	6.80 (2.50, 12.43)	7.29 (3.91, 7.29)	<0.001^c^
Serum creatinine, mg/dL	33/34/44	0.6 (0.6, 0.8)	0.7 (0.6, 0.9)	1.25 (1.00, 1.90)	<0.001^a^
eGFR, mL/min/1.73 m^2^	33/34/44	120.66 (24.09)	111.13 (25.90)	54.74 (24.97)	<0.001^a^

Mean (SD) or median (25th percentile, 75th percentile) shown for continuous variables (normally distributed or non-normally distributed, respectively). Frequency (percentage) shown for categorical variables. Statistical significance defined with a *p* < 0.05. IU: International Unit. eGFR: estimated glomerular filtration rate

Group categorization: control group defined as non-diabetic patients with eGFR ≥ 90 mL/min/1.73 m^2^, DM group defined as diabetic patients with eGFR ≥ 90 mL/min/1.73 m^2^ and CKD group defined as diabetic patients with eGFR < 90 mL/min/1.73 m^2^

N_1_ = Control

N_2_ = DM

N_3_ = CKD

^a^CKD statistically different than DM and control

^b^DM statistically different than control

^c^Control statistically different than DM and CKD

^d^Control statistically different than CKD

**Fig. 2:**
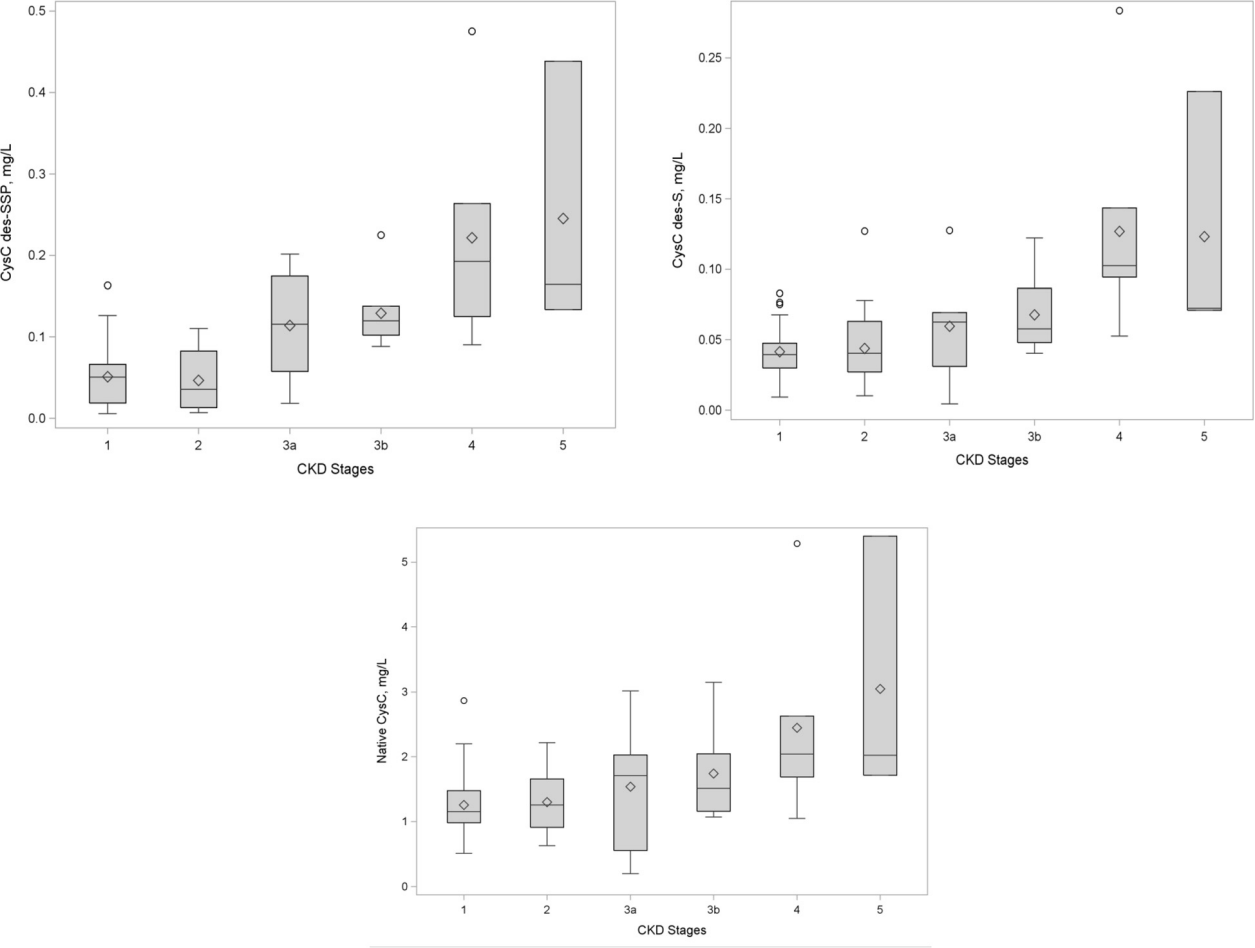
Distribution of CysC proteoforms concentrations across CKD stages in diabetic patients with and without CKD. There are 5 CKD stages based on estimated GFR. Stage 1 is define by eGFR ≥ 90 mL/min/1.73 m^2^ (*n* = 34), stage 2 is defined by 60 ≤ eGFR ≤ 89 mL/min/1.73 m^2^ (*n* = 21), stage 3a is defined by 45 ≤ eGFR ≤ 59 mL/min/1.73 m^2^ (*n* = 6), stage 3b is defined 30 ≤ eGFR ≤ 44 mL/min/1.73 m^2^ (*n* = 7), stage 4 is defined by 15 ≤ eGFR ≤ 29 mL/min/1.73 m^2^ (*n* = 7), and stage 5 is defined by eGFR < 15 mL/min/1.73 m^2^ (*n* = 3). All CysC proteoforms significantly increased across the advanced CKD stages (group ANOVA test). Using pairwise comparisons, des-SSP: stages 1-3b, 1–5, 1–4, 2-3b, 2–4, 2–5; des-S: stages 1–4, 1–5, 2–4, 3a-4; native: stages 1–5 and 1–4 were statistically different. These data suggest that des-SSP and des-S CysC proteoforms increase more robustly by CKD stage compared with native CysC concentrations

Using mass spectrometric immunoassay we were able to identify several CysC proteoforms (Table 2). An example of a mass spectra of CysC from a plasma sample of a control participant is shown in Fig. 1. Native CysC (representing sum of native and 3Pro-OH CysC), CysC-des-S (sum of des-S CysC and des-S 3Pro-OH CysC), and CysC-des-SSP concentrations were greater in participants with CKD compared to participants without CKD (38–77 % greater CysC proteoform concentrations, *p* < 0.01). Greater increases in truncated CysC were observed across the different CKD stages compared to native CysC concentrations (Fig. 2). The distribution of CysC proteoforms across the three groups is summarized in Table 3. Native CysC concentrations were 1.4 fold greater in CKD compared to DM group (*p* = 0.02) and 1.5 fold greater in CKD compared to the control group (*p* = 0.001). CysC des-S concentrations were 1.55 fold greater in CKD compared to the DM group (*p* = 0.002) and 1.9 fold greater in CKD compared to the control group (*p* = 0.0002). CysC des-SSP concentrations were 1.8 fold greater in CKD compared to the DM group (*p* = 0.008) and 1.52 fold greater in CKD compared to the control group (*p* = 0.002).

**Table 2:** Cystatin C proteoforms detected by MSIA

Theoretical m/z	Observed m/z	Proteoform	Label
13,071.833	13,072.684	Cystatin C lacking N-terminal tripeptide (SSP-)	des-SSP CysC
13,256.027	13,256.897	Cystatin C lacking one N-terminal serine residue (S-)	des-S CysC
13,272.027	13,272.649	Hydroxylated cystatin C (on 3-Pro residue) lacking N-terminal S-residue^a^	des-S 3Pro-OH CysC
13,343.105	13,344.169	Full length, unmodified cystatin C	native CysC
13,359.105	13,359.246	Hydroxylated cystatin C in position 3 on proline (Pro) residue^a^	3Pro-OH CysC
18,276.162	18,276.500	Beta lactoglobulin (internal reference standard)	BL

CysC exists in multiple forms. The three main CysC variants are one containing hydroxyproline and 2 truncated variants; one missing N-terminal serine, and one lacking three N-terminal residues. *MSIA*: Mass Spectrometric Immunoassay. *BL*: Beta lactoglobulin is the internal standard

^a^The hydroxylated variants were co-integrated with their originating proteoforms, therefore were not individually correlated with the clinical parameters

**Table 3:** Distribution of CysC proteoforms concentrations by DM and CKD groups

	N1/N2/N3	Control	DM	CKD	*p*-value
Native CysC (mg/L)	33/34/44	1.09 (0.71,1.35)	1.15(0.97,1.48)	1.59 (1.07, 2.02)	<0.01^a^
CysC des-S (mg/L)	33/34/44	0.03 (0.025,0.046)	0.039 (0.03,0.05)	0.059 (0.035,0.08)	<0.01^b^
CysC des-SSP (mg/L)	33/34/44	0.06 (0.04,0.09)	0.053 (0.02, 0.071)	0.094(0.035, 0.14)	<0.01^c^

Median (25th percentile, 75th percentile). Statistical significance defined with a *p* < 0.02. Groups compared with ANOVA followed by Tukey’s pairwise comparisons

N_1_ = Control

N_2_ = DM

N_3_ = CKD

^a^Native CysC

In DM vs. controls, *p* = 0.6

In CKD vs. DM, *p* = 0.02

In CKD vs. controls, *p* = 0.001

^b^CysC des-S

In DM vs. controls, *p* = 0.8

In CKD vs. DM, *p* = 0.002

In CKD vs. controls, *p* = 0.0002

^c^CysC des-SSP

In DM vs. controls, *p* = 0.9

In CKD vs. DM, *p* = 0.008

In CKD vs. controls, *p* = 0.002

To understand the relation of CysC proteoforms with CKD, the concentrations of these proteoforms were correlated with eGFR. Increases in CysC proteoforms were negatively correlated with eGFR. This linear association between CysC proteoform concentrations and eGFR was significant after adjusting for age, sex and BMI, as summarized in Table 4. However, subgroup analysis indicated that this association of CysC proteoforms and eGFR was driven by the CKD group.

**Table 4:** Association of CysC concentrations with eGFR

		Combined sample		Control		DM		CKD	
	N/N_1_/N_2_/N_3_	Regression coefficient Beta (SE)	*P*-value	Regression coefficient Beta (SE)	*P*-value	Regression coefficient Beta (SE)	*P*-value	Regression coefficient Beta (SE)	*P*-value
Total CysC	107/33/34/40	−16.40 (17.62)	<0.0001	2.47 (10.82)	0.82	−4.96 (7.66)	0.52	−10.09 (3.36)	<0.01
CysC des-SSP	107/33/34/40	−245.96 (44.41)	<0.0001	−83.46 (106.80)	0.44	−132.88 (116.49)	0.26	−177.17 (29.44)	<0.001
CysC des-S	107/33/34/40	−486.92 (92.06)	<0.0001	−375.06 (310.95)	0.24	−100.46 (213.44)	0.64	−281.68 (72.88)	<0.001
Native CysC	107/33/34/40	−16.40 (4.03)	<0.001	4.32 (11.60)	0.71	−4.86 (8.18)	0.56	−10.24 (3.86)	0.01

Linear regression with eGFR as the dependent variable and total CysC, CysC des-SSP, CysC des-S, native CysC concentrations as the independent variables adjusted for age, sex and BMI. Type Ι error rate = 0.02 adjusted for multiple comparisons

N = number of subjects in combined sample (control + DM + CKD)

N_1_ = Control

N_2_ = DM

N_3_ = CKD

The concentrations of truncated proteoforms of CysC had a stronger correlation with eGFR than native CysC proteoform. The regression coefficients explaining the correlation between CysC proteoforms and eGFR were larger for truncated CysC proteoforms than the native CysC proteoform. The truncated CysC proteoforms correlated with eGFR independent of the native CysC. The partial correlation of native CysC and CysC truncations with eGFR demonstrated a significant inverse correlation between CysC des- SSP and CysC des-S concentrations and eGFR (*r* = −0.28, *p* = 0.003, and *r* = −0.24, *p* = 0.01, respectively). The partial correlation of native CysC with eGFR was not significant (*r* = 0.15, *p* = 0.12). Therefore, truncated CysC proteoforms were associated with eGFR independent of native CysC; highlighting the importance for measuring the truncated proteoforms in CKD patients.

To examine the relation of CysC truncations with albuminuria, the distribution of CysC proteoforms was analyzed in the combined sample (*n* = 104) where urine microalbumin was measured. The three categories of albuminuria were defined as no albuminuria (urine microalbumin < 30 mcg/mg creatinine), micoalbuminuria (urine microalbumin 30–300 mcg/mg creatinine) and macroalbuminuria (urine microalbumin > 300 mcg/mg creatinine). Eight patients had clinical albuminuria, 18 patients had micoalbuminuria, and 78 participants presented with no evidence of albuminuria. Participants with albuminuria had greater amounts of plasma CysC proteoforms (*p* < 0.05) compared to participants with normal albuminuria (Table 5). CysC des-SSP concentrations in the macroalbuminuria group were 1.57 fold greater compared to the normoalbuminuria group (*p* = 0.02). CysC des-S concentrations in the macroalbuminuria group were 1.56 fold greater compared to the normoalbuminuria group (*p* = 0.05).

**Table 5:** Distribution of CysC proteoforms concentrations by albuminuria groups

	N1/N2/N3	Normoalbuminuria	Microalbuminuria	Macroalbuminuria	*p*-value
Native CysC (mg/L)	78/18/8	1.18 (0.51)	1.56 (0.57)	1.70 (0.68)	0.02^a^
CysC des-S (mg/L)	78/18/8	0.041 (0.025)	0.053 (0.030)	0.06 (0.019)	0.003^b^
CysC des-SSP (mg/L)	78/18/8	0.061 (0.047)	0.096(0.071)	0.096 (0.048)	0.01^c^

Mean (SD). Statistical significance defined with a *p* < 0.02. Groups compared with ANOVA followed by Tukey’s pairwise comparisons

N_1_ = number of participants with normoalbuminuria

N_2_ = number of participants with microalbuminuria

N_3_ = number of participants with macroalbuminuria

^a^Native CysC

In macro vs. normoalbuminuria, *p* = 0.03

In macro vs. microalbuminuria, *p* = 0.8

In micro vs. normoalbuminuria, *p* = 0.03

^b^CysC des-S

In macro vs. normoalbuminuria, *p* = 0.05

In macro vs. microalbuminuria, *p* = 0.6

In micro vs. normoalbuminuria, *p* = 0.2

^c^CysC des-SSP

In macro vs. normoalbuminuria, *p* = 0.16

In macro vs. microalbuminuria, *p* = 0.99

In micro vs. normoalbuminuria, *p* = 0.02

## Discussion

Our findings demonstrate that CysC proteoforms are greater in concentrations in participants in the CKD + DM group compared to the DM and control groups. Native CysC and CysC truncations (CysC des-SSP and CysC des-S) were inversely correlated with eGFR and this persisted after adjusting for age, sex and BMI. CysC proteoforms concentrations were also significantly greater in the setting of urine microalbuminuria. The association with eGFR was stronger with the truncated proteoforms than native CysC and was only significant in participants with both CKD and DM. These findings highlight the importance of measuring truncated CysC proteoforms.

Total CysC was shown previously to be a specific predictor of eGFR [8, 10]. The lack of correlation of CysC proteoforms with eGFR in the control and DM groups was noteworthy. The MDRD Study equation to estimate GFR is best in the lower ranges of GFR [18]. GFR estimates from the MDRD Study equation greater than 60 mL/min/1.73 m^2^ underestimate measured GFR, and may lead to misdiagnosis and misclassification of CKD in individuals with mild renal insufficiency [19, 20]. This limitation highlights the need of new CKD biomarkers to capture early disease risk.

The mechanism of CysC truncations is not well understood. The N-terminal residues of CysC were found to be important in determining the specificity of CysC with cysteine proteinases [21]. It is known that leucocyte elastase cleaves the Val10-Gly11 bond of cystatin C [22], and cathepsin L cleaves the Gly11-Gly12 bond [23]. The NMR structure of CysC shows that the N-terminal fragment is unstructured and highly mobile [24]. The N-terminus also contains the binding site for the cysteine proteinases, and when removed through truncations, the inhibition of proteinases is drastically reduced due to the decreased affinity [21]. Therefore, it is likely that the truncated des-S and des-SSP CysC proteoforms exhibit reduced biological activity. Our data suggest that CysC truncations can be markers of disease severity and used as more specific measures of CKD than native CysC. Therefore, evaluating changes in CysC truncations can be a useful indicator of CKD progression.

The strength of this study lies in the simple MS-based quantitative proteomics approach to assess CysC proteoforms, that is both high throughput and accurate. There are some limitations to this study. MSIA was not able to resolve signals from hydroxylated CysC (3Pr-OH) from native CysC at baseline due to the small mass difference among these proteoforms. A previous study demonstrated CysC and truncations levels can fluctuate daily by as much as 2 fold within the normal range [13], which could bias our findings given our limited sample size. Another limitation is the small sample size of the subgroups with advanced CKD, stages 4 and 5. Our findings warrant an investigation of Cystatin C proteoforms in larger CKD studies with measured GFR to discriminate the capacity of these proteoforms versus eGFR in predicting GFR decline.

## Conclusions

We conclude that CysC proteoforms are increased in patients with DM and CKD. Truncated proteoforms, independent of native CysC, associate with eGFR. Future studies are needed to further investigate the relation between CysC proteoforms in early renal disease and for their utility as biomarkers of CKD progression.

## Additional file

Additional file 1: Figure S1. Example a) standard curve for determination of CysC concentration and b) corresponding mass spectra from CysC standards obtained with MSIA. (ZIP 317 kb)
