# Supplementary figures and images for: The association of plasma cystatin C proteoforms with diabetic chronic kidney disease
Source: Proteome Sci. 2016 Mar 25;14:7. doi: 10.1186/s12953-016-0096-7 (PMC4807542; doi:10.1186/s12953-016-0096-7)

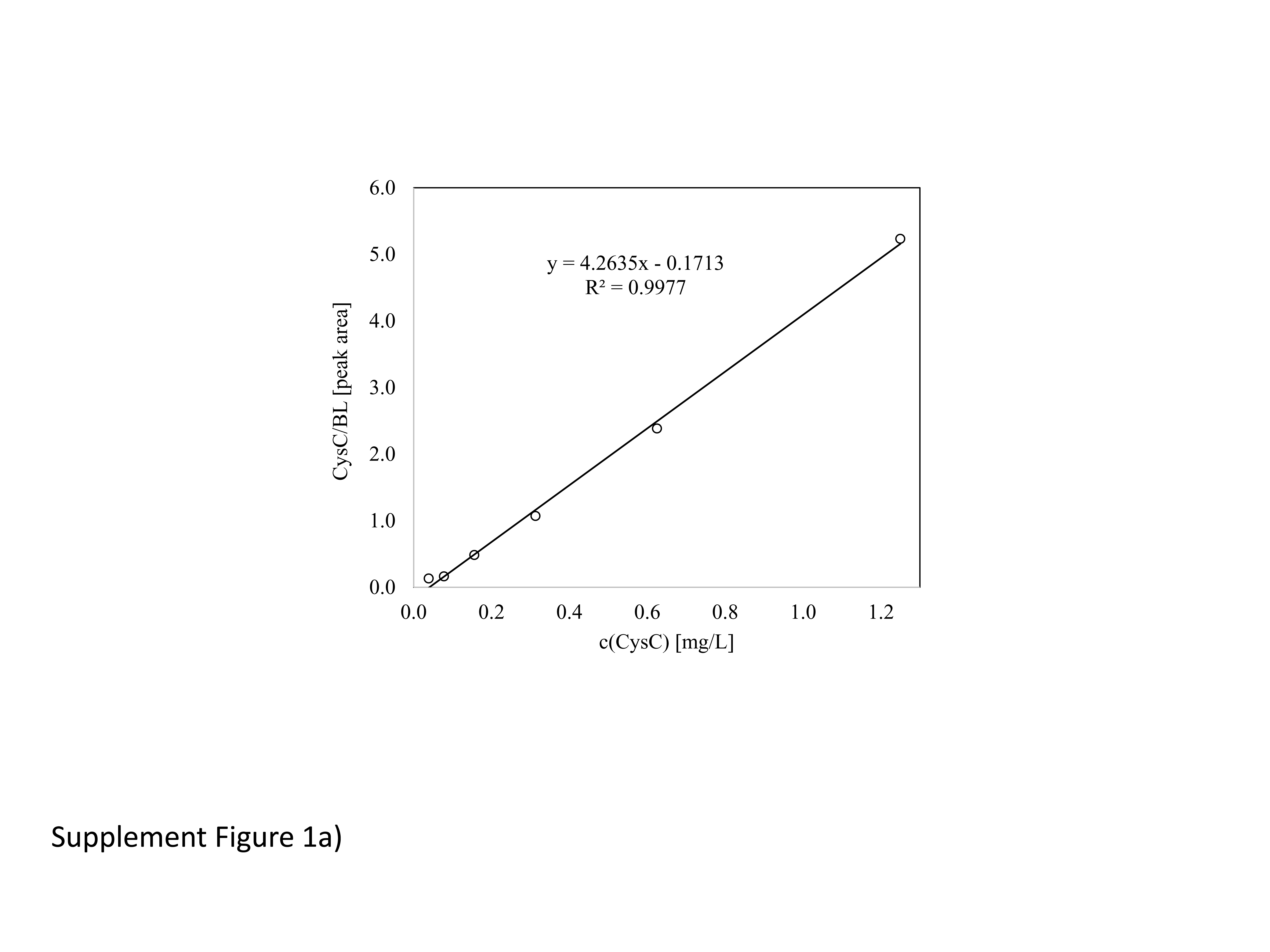

Supplement: Additional file 1: Figure S1. — Example a) standard curve for determination of CysC concentration and b) corresponding mass spectra from CysC standards obtained with MSIA. (ZIP 317 kb) [file 12953_2016_96_MOESM1_ESM.zip › S Fig 1a.tif]

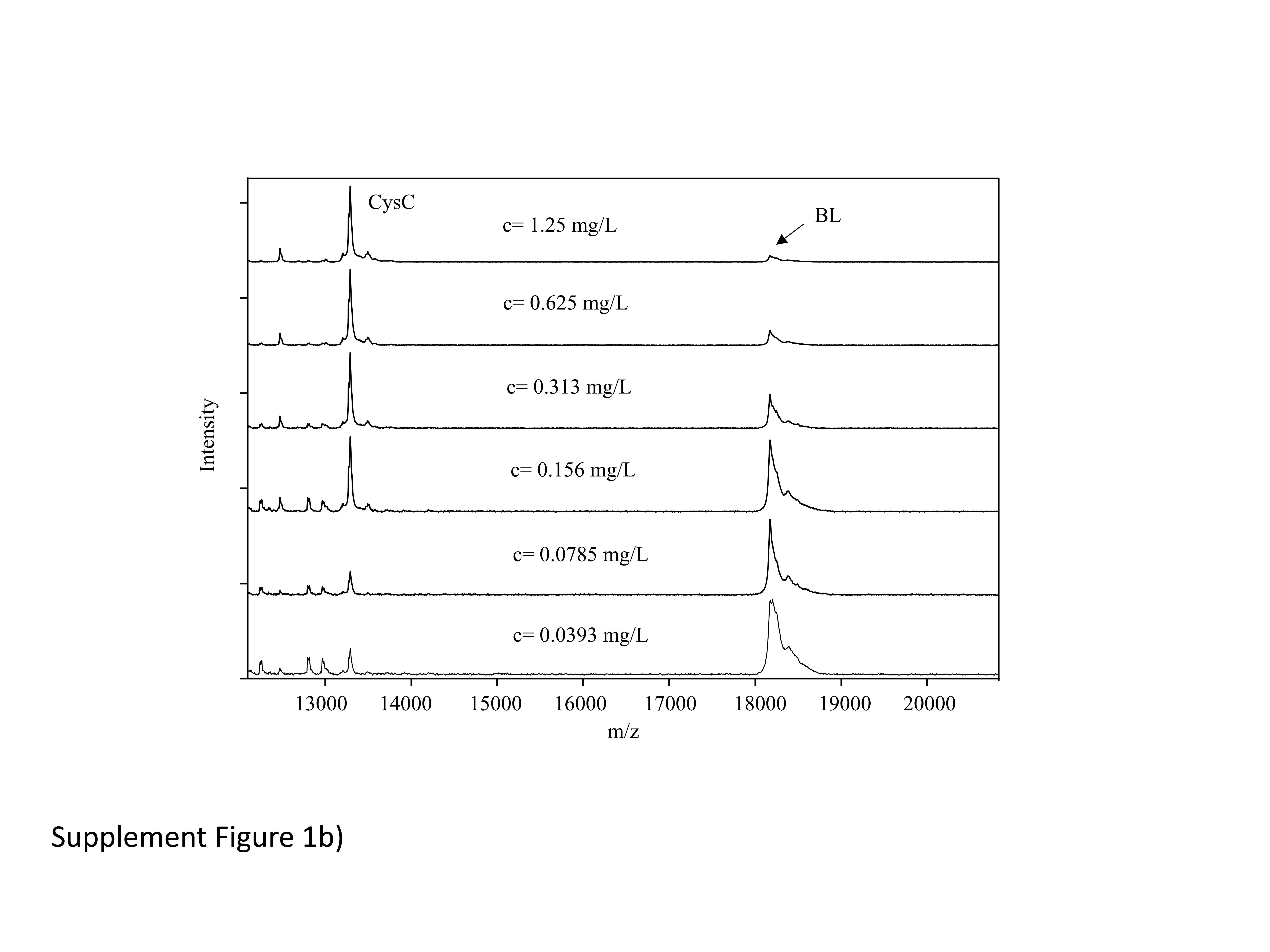

Supplement: Additional file 1: Figure S1. — Example a) standard curve for determination of CysC concentration and b) corresponding mass spectra from CysC standards obtained with MSIA. (ZIP 317 kb) [file 12953_2016_96_MOESM1_ESM.zip › S Fig 1b.tif]
